# The ethanolic extract of *Aralia continentalis* ameliorates cognitive deficits via modifications of BDNF expression and anti-inflammatory effects in a rat model of post-traumatic stress disorder

**DOI:** 10.1186/s12906-018-2417-0

**Published:** 2019-01-08

**Authors:** Bombi Lee, Riwon Hong, Pooreum Lim, Daeun Cho, Mijung Yeom, Sanghyun Lee, Ki Sung Kang, Sang Cheon Lee, Insop Shim, Hyejung Lee, Dae-Hyun Hahm

**Affiliations:** 1 Acupuncture and Meridian Science Research Center, College of Korean Medicine, Kyung Hee University, Seoul, 02447 Republic of Korea; 2 The Graduate School of Basic Science of Korean Medicine, College of Korean Medicine, Kyung Hee University, Seoul, 02447 Republic of Korea; 3 Department of Integrative Plant Science, Chung-Ang University, Anseong, 17546 Republic of Korea; 4 College of Korean Medicine, Gachon University, Seongnam, 13120 Republic of Korea; 5 Imsil Cheese & Food Research Institute, Imsil, 55918 Republic of Korea; 6 Department of Physiology, College of Medicine, Kyung Hee University, Seoul, 02447 Republic of Korea; 7 BioNanocomposite Research Center, Kyung Hee University, Seoul, 02447 Republic of Korea

**Keywords:** *Aralia continentalis*, Post-traumatic stress disorder, Memory, Brain-derived neurotrophic factor, Pro-inflammatory cytokines

## Abstract

**Abstract:**

**Background:**

Post-traumatic stress disorder (PTSD) is a disease associated with that the experience of traumatic stress. The traumatic experience results in the development of a prolonged stress response that causes impaired memory function and increased inflammation in the hippocampus. Currently, antidepressants are the only approved therapy for PTSD. However, the efficacy of antidepressants in the treatment of PTSD is marginal. The ethanol extract of *Aralia continentalis* (AC) is traditionally used in oriental medicine, and has been showed to possess pharmacological properties, including anti-inflammatory, anti-cancer, anti-atherosclerotic, and anti-diabetic effects. Nevertheless, the effects of AC on cognitive memory and its mechanism of action in PTSD remain unclear. Given the necessity of further treatment options for PTSD, we investigated the effect of AC on the spatial cognitive impairment caused by single prolonged stress (SPS) in a rat model of PTSD.

**Methods:**

Male rats were treated with various intraperitoneal (i.p.) doses of AC for 21 consecutive days after inducing chronic stress with the SPS procedure.

**Results:**

Cognitive impairment caused by SPS were inhibited after treatment with 100 mg/kg AC, as measured by the Morris water maze test and an object recognition test. Additionally, AC treatment significantly alleviated memory-related decreases in brain-derived neurotrophic factor (BDNF) mRNA and protein levels in the hippocampus. Our results suggest that AC significantly inhibited the cognitive deficits caused by SPS via increased expression of pro-inflammatory cytokines, including tumor necrosis factor-α and interleukin-6, in the rat brain.

**Conclusions:**

AC reversed the behavioral impairments and inflammation triggered by SPS-derived traumatic stress and should be further evaluated as a potential therapeutic drug for PTSD.

## Background

Post-traumatic stress disorder (PTSD) is a psychiatric disorder that can develop after witnessing a life-threatening traumatic event or experiencing a crime, war, natural catastrophe, terrorist assault, or dangerous car accident [1, 2]. In PTSD, stimuli associated with the traumatic event can lead to re-experiencing the event in the form of recurrent nightmares and flaskbacks [1, 3], and feelings of intense fear, helplessness, and horror. Ultimately, this leads to efforts to actively avoid such reminders [4] and the formation of highly aversive and intrusive memories related to the trauma that are resist to extinction [2, 5]. Thus, cognitive and memory deficits are frequently observed in patients with PTSD [2, 6]. Although the past decade has seen significant advances in the understanding of the pathogenesis of PTSD, the underlying pathology of the condition is not yet fully understood [3].

To study the behavioral and biological mechanisms of PTSD, the single prolonged stress (SPS) animal model offers many advantages and is thus widely used [7]. Animals in which SPS was induced predictable and continuous features associated with trauma, such as anxiety, depression-like behavior, impaired fear extinction, changes in hypothalamic-pituitary-adrenal (HPA) axis function [8], and increased apoptosis in the hippocampus; all of these are psychological symptoms of PTSD [9]. Hippocampal neurons, which play a major role in the cognition and memory alterations caused by SPS [2, 10], and also involved in deficits in spatial memory [10, 11].

Moreover, patients with PTSD may show impaired neurogenesis in, and development of, the hippocampus [12]. Hippocampal brain-derived neurotrophic factor (BDNF) and cAMP-response element-binding protein (CREB) play important roles in the pathology of PTSD [2, 13]. Furthermore, it has been demonstrated that proinflammatory cytokine production is stimulated in patients that experience traumatic stress and results in the disruption of neuronal circuitry [14, 15]. In fact, the drugs used to treat PTSD have pharmacological properties that affect the psychosocial stress that characterizes the disorder. For example, the antidepressant fluoxetine (FLX) has been showed to exert anti-inflammatory effects [3].

Another potential pharmacological treatment for PTSD is use of selective serotonin reuptake inhibitors (SSRIs), which are thought to play an important role in the course of the disease [16]. Numerous pharmacological therapies are available for treatment of PTSD symptomatology. However, extensive side effects are associated with these drugs [2]. Therefore, there is a critical need for a novel therapeutic approach for PTSD treatment and management [17]. As a result, much attention has been paid to novel natural medicines that may be safer than pharmaceutical agents for long-term treatment of PTSD [18].

*Aralia continentalis* Kitagawa (AC), also known as “Dokwhal” in Korea, is a folk medicine widely used in East Asia [19] to alleviate pain associated with headache, rheumatism, lumbago, and lameness [20]. Several constituents of AC root extract have been identified as having sedative, antifungal, analgesic, antioxidant, and anti-inflammatory action [21, 22]. The dichloromethane fractions and methanol extract of these plant materials have been found to attenuate interleukin-8 (IL-8) production via lipopolysaccharide (LPS)-treated peritoneal macrophages [23]. Furthermore, the methanol extract of AC exhibits antinociceptive effects against Freund’s adjuvant-induced pain in rats [24]. The extract has also been found to protect against cartilage degradation and inhibit apoptosis, suggesting that it has the potential to inhibit osteoarthritis [25]. AC may also significant suppress prostaglandin E2 and nitric oxide (NO) production in LPS-induced RAW 264.7 cells [20, 26]. The AC root extract also markedly inhibits *Streptococcus mutans* (*S. mutans*) adherence to saliva-coated hydroxyapatite beads [19], and recent studies have found that a crude extract of AC inhibited *S. mutans* bacterial growth, acid production, adherence to hydroxyapatite beads, and biofilm formation [19, 27].

Other experimental data have documented that ethnopharmacological effects of ethanol and methanol extracts of the AC root on the inflammatory response. AC extract has been found to inhibit the activity of cyclooxygenase-2 (COX-2) and inducible NO synthase expression, as well as block the nuclear factor kappa-light-chain-enhancer of activated B cells [20, 28]. The ethanol extract of AC stored in the herbarium of the Imsil Cheese and Food Research Institute in Korea is among the 68 accepted species of the *Aralia genus*.

Although a brief report on the anti-inflammatory effects of AC has been published [20, 26], it is not clear whether the ethanol extract of AC improves SPS-induced recognition and spatial memory deficits as measured by the Morris water maze (MWM) test and an object recognition test (ORT), respectively. Furthermore, we examined the relationship between stress-induced learning and memory deficits and CREB and BDNF expression, and the effects of such stress on inflammation in the hippocampus, with the aim of developing a novel treatment or trauma-associated disorders such as PTSD.

## Methods

### Animals

Eight week-old male Sprague-Dawley (SD) rats (Samtako Animal Co., Seoul, Korea), weighing 200–230 g were used in all examinations. Rats were housed in pairs for 1 week prior to the start of the experiment, and were maintained on a 12/12 h light-dark cycle and fed ad libitum throughout the duration of testing. All methods and procedures were approved by the Animal Care and Use Committee of Kyung Hee University (KHUASP (SE)-15–115). All procedures were executed according to the Guide for the Care and Use of Laboratory Animals.

### Preparation of an ethanol extract of *Aralia continentalis* from Imsil

Dried roots of *A. continentalis* Kitag. (AC) were supplied by the Imsil Cheese and Food Research Institute (ICFRI, Korea) and verified by Dr. K. Choi (Korea National Arboretum, Korea). A voucher specimen was deposited at the herbarium of ICFRI (No. D201505ACI). AC was extracted and purified using the method of Hong et al. [29]. The extract was suspended in distilled water for future work.

### HPLC analysis of AC extract

**Fig. 1:**
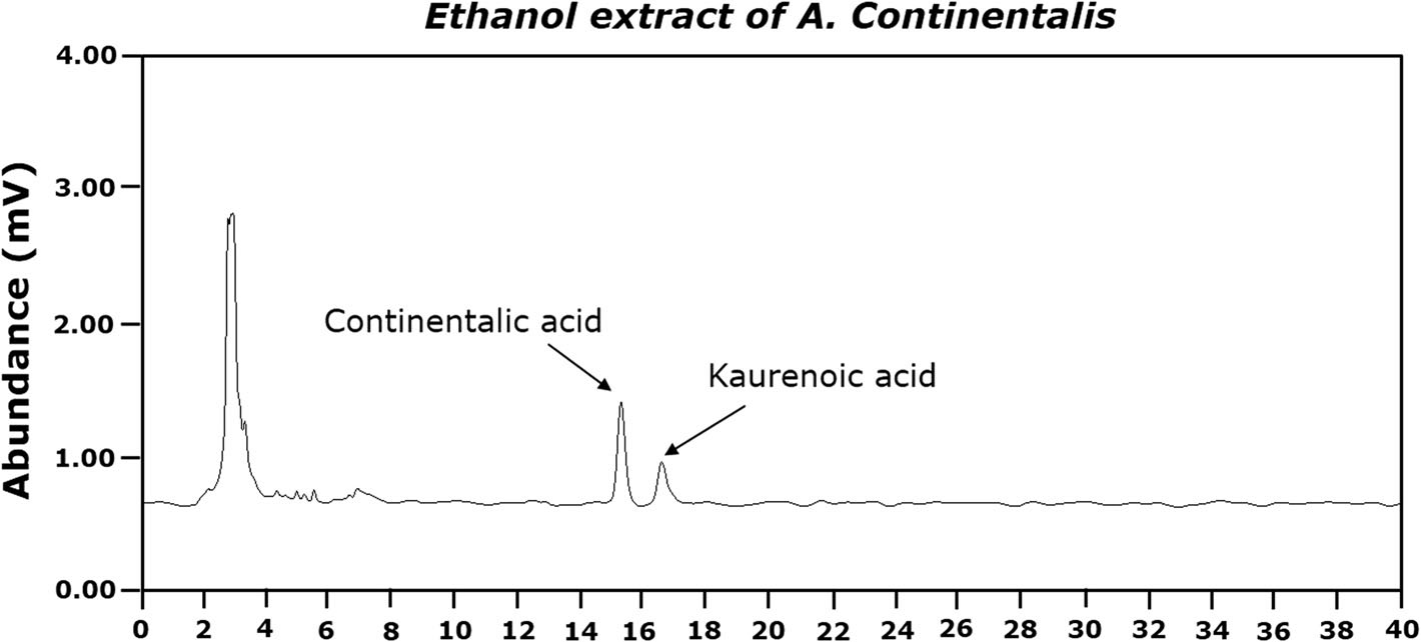
High performance liquid chromatography (HPLC) chromatogram of AC. Phytochemical analysis was performed using HPLC as described in the Materials and Methods section. Authentic standard of continentalic acid (7-oxo-ent-pimara-8,15-diene-19-oic acid) and kaurenoic acid (en-kaura-16-en-19-oic-acid) were used, and their contents in the AC extract were determined to be 12.097 ± 0.200 and 3.378 ± 0.253 mg/g extract, respectively

The concentration of AC in the samples was assayed using high performance liquid chromatography (HPLC) coupled with an ultraviolet visible detector, as previously described [29]. A representative HPLC chromatogram of the 50% ethanol extract of AC is presented in Fig. 1. Kaurenoic acid (KA, ent-kaura-16-en-19-oic acid) and continentalic acid (CA, (−)-pimara-8(14), 15-diene-19-oci acid) were purchased from the National Development Institute of Korean Medicine (Gyeongsangbuk-do, Korea) and ChemFaces (Wuhan, Hubei, China), respectively. The concentrations of KA and CA were calculated using calibration curves established from the standard compounds. The control levels of KA and CA in the ethanol extract of AC were 12.097 ± 0.200 and 3.378 ± 0.253 (mg/g extract), respectively.

### Single prolonged stress

Rat were exposed to SPS for 14 successive days, as described previously [30]. Briefly, rats were restrained for 2 h, followed immediately by a 20 min forced swim. The rats were allowed to recover for 15 min in a home cage, and then exposed to ether until they were unconscious. For sensitization testing, rats were left undisturbed in their home cages for 14 days to allow PTSD-like symptoms to manifest without disturbances, excluding necessary disturbances to monitor their health condition and replenish food and water as necessary [30].

### AC administration

**Fig. 2:**
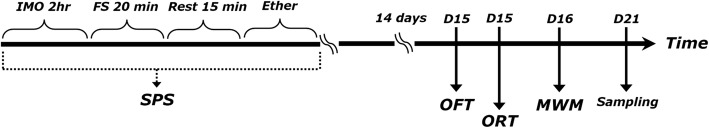
Experimental protocol of single-prolonged stress (SPS)-induced memory impairment and subsequent treatment with *A. continentalis* (AC)(B). Groups of seven rats were used for each experimental condition. OFT; open field test, ORT; object recognition task, MWM; Morris water maze test, IHC; immunohistochemistry

In the current study, the criterion doses of AC in the rat, and considerations relating the long-term use of the medication, were based on a former study [24]. AC (20, 50 and 100 mg/kg body weight) and the positive control drug fluoxetine hydrochloride (10 mg/kg, FLX, Sigma-Aldrich Chemical Co., St. Louise, MO, USA) were injected intraperitoneally (i.p.) for 21 days after subjecting the rats to SPS. AC and FLX were dissolved in 0.9% saline solution prior to injection. All drugs were freshly prepared before every experiment. The experimental schedule is shown in Fig. 2.

### Object recognition test

Hippocampus-dependent non-emotional memory was examined using an ORT, as described previously [31]. Briefly, each rat was guided individually into a rectangular container (45 × 45 × 25 cm). The objects to be discriminated were two similar-looking wooden block toys (the familiar objects; A1 and A2), which were heavy enough so that rats were not able to move them. A different shaped and colored wooden block toy was used as the novel object, B.

The experiment was performed in three phases; habituation, training, and testing. In the habituation phase, the rats were pre-exposed to the rectangular container for 10 min. After habituation, rats were positioned inside the box with two similar objects (A1 and A2) and were allowed to explore the objects for 5 min. During the test phase, rats were allowed to explore one new object (B) and one of the old objects for 5 min. The sniffing time for the novel and familiar object was measured. The discrimination index was calculated as: (time spent on novel object – time spent on familiar object)/(time spent on novel object + time spent on familiar object).

### Morris water maze test

The rat’s spatial learning and memory abilities were evaluated using the MWM, performed as described previously [32]. All six groups of rats were trained and tested in the MWM test for the purpose of measuring spatial memory after ORT. The water maze consisted of a large circular pool (2.0 m in diameter, 60 cm in height, filled to a depth of 45 cm with water at 22 ± 2 °C). The pool was divided into four equal hypothetical quadrants and included a hidden circular platform in one quadrant (15 cm in diameter). The position of the platform was unaltered throughout the training session. The MWM trials were recorded by a video camera mounted on the ceiling and the data were analyzed using a tracking program (S-MART: PanLab Co., Barcelona, Spain). The MWM test includes two main parts: the place navigation test (wherein the rats must reach a hidden platform) and the retention test. For the place navigation test, the study was performed in the dark and rats completed three training trials per day for 5 consecutive days, with the aim of locating a submerged platform using visual cues installed around the room. The retention test was performed on day 6, after removing the escape platform, and the probe trials were conducted for 60 s. In the probe trial, the swimming speed, frequency of platform crossing, time taken, and swimming path length in the target quadrant were measured.

### Open field test

Before completion the MWM trial, the rats were exposed to the open field test (OFT), as described previously [30]. Each rat was individually placed in a rectangular container (60 × 60 × 30 cm) in the room. This results in the best contrast of the white rats against the dimly lit background of the room, which was equipped with a video camera above its center point. The distance and speed of movements were monitored by a computerized video-tracking system using the S-MART program (PanLab Co) to track locomotor activities. The number of rearing events for each rat was also recorded to analyze locomotor activity in the OFT.

### Measurement of corticosterone, BDNF, CREB and proinflammatory markers

Animals were anesthetized for euthanasia with the following reagents: isoflurane (Hanlim Pharm. Seoul, Korea), and sodium pentobarbital (Hanlim Pharm). Isoflurane was administered by inhalation at a overdose of 4% with oxygen as a carrier gas for 5 min in a sealed chamber at a pressure of 6 psi by using inhalation anesthesia apparatus (IWOO Scientific Corporation, Seoul, Korea) at room temperature to induced euthanasia according to the Institutional Animal Care and Use Committee (IACUC) Guidelines for the Euthanasia of Korea. Pentobarbital was injected intraperitoneally at a dose of 100 mg/kg body weight.

At 21 days after inducing SPS, corticosterone (CORT) in the plasma, interleukin (IL)-6, tumor necrosis factor-α (TNF-α), CREB, and BDNF concentrations in the brain, were assayed using previously described methods [30]. Plasma (*n* = 4/group) was collected via the abdominal aorta. The hippocampus (*n* = 4/group) was quickly dissected from each rat brain in random order. Levels of CORT, CREB, BDNF, TNF-α, and IL-6 were assessed by competitive enzyme-linked immunosorbent assay (ELISA) using a CORT antibody (CORT ELISA kit; Novus Biologicals, LLC., Littleton, CO, USA), a CREB antibody (CREB ELISA kit; Thermo Fisher Scientific, Waltham, MA, USA), a BDNF antibody (BDNF ELISA kit; R&D Systems, Minneapolis, MN, USA), a TNF-α antibody (TNF-α ELISA kit; Abcam, Cambridge, UK), and an IL-6 antibody (IL-6 ELISA kit; Abcam).

### Total RNA isolation and RT-PCR analysis

The expression levels of CREB, BDNF, IL-6 and TNF-α mRNA were measured by reverse transcription**-**polymerase chain reaction (RT**-**PCR), as described previously [30]. The rats were anesthetized via inhalation of isoflurane (4%) and decapitated with a guillotine; the brains were then harvested. A coronal section of the hippocampus was dissected from the brain. Total RNA was isolated from homogenates using the miRNeasy Kit (Qiagen, Hilden, Germany) according to the manufacturer’s protocol. cDNA was synthesized from total RNA using reverse transcriptase (Takara Bio, Otsu, Japan), and then amplified by PCR using Taq DNA polymerase (Takara, Kyoto, Japan) on a thermal cycler (MJ Research, Watertown, MA, USA). cDNA expression levels were eventually determined by adjusting each band intensity to that of the glyceraldehyde 3-phosphate dehydrogenase (GADPH) control.

### Immunohistochemistry

Immunohistochemistry was also conducted to evaluate the BDNF level in the hippocampus, as described previously [30]. Briefly, three rats from each group were anesthetized deeply via inhalation of isoflurane (4%) and their brain tissues were collected. Free-floating tissue sections were incubated overnight with primary rabbit anti-BDNF antibody (1:200 dilution, Cell Signaling Technology, Boston, MA, USA) and the sections were then incubated for 2 h at room temperature with secondary antibodies (1:200 dilution; Vector Laboratories Co., Burlingame, CA, USA). Next, the sections were incubated with avidin-biotin-peroxidase complex (Vector Laboratories) for 1 h at room temperature and then in a solution containing 3,3′-diaminobenzidine (DAB; Sigma-Aldrich) and 0.03% hydrogen peroxide for 1 min. The slides were viewed at 200× magnification, and the number of BDNF labeled cells in the hippocampus was determined.

### Statistical analysis

All data are presented as means ± SEM. Statistical differences among groups were identified by analysis of variance (ANOVA) and with Tukey’s post hoc tests, conducted in SPSS software (ver. 13.0; SPSS, Inc., Chicago, IL, USA). *P*-values of < 0.05 were considered statistically significant.

## Results

### Effect of AC on SPS-induced alterations of plasma CORT levels

**Fig. 3:**
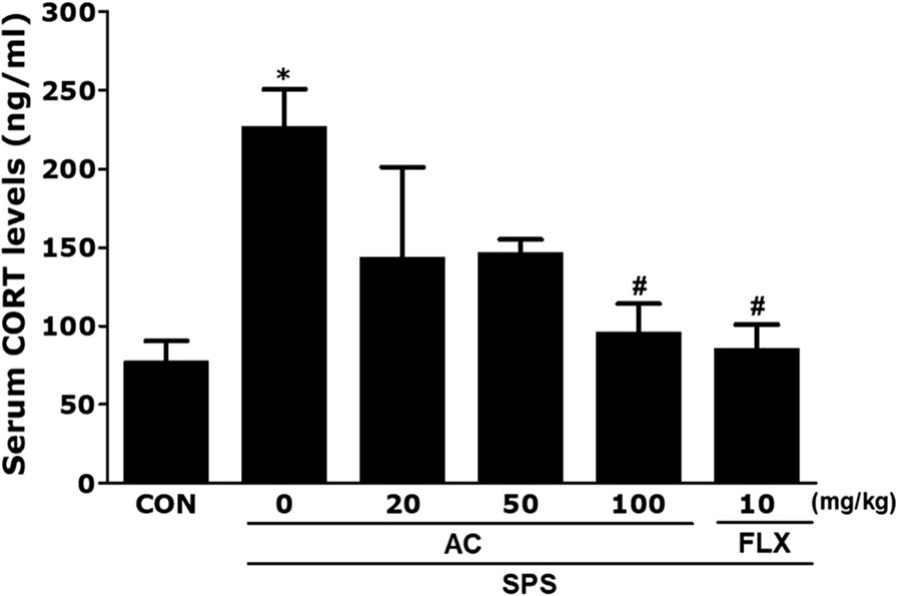
Effects of AC on serum corticosterone (CORT) levels in rats with SPS-induced memory impairment. Results were analyzed by enzyme-linked immunosorbent assay (*n* = 4/group). *p < 0.05 vs. the CON group, #*p* < 0.05 vs. the SPS group

To assess the effects of the SPS regimen and AC on the stress hormone system, we measured the levels of plasma CORT. ELISA revealed that SPS treatment significantly increased the plasma CORT level in rats, by 294.15%, compared to the saline-treated group after 21 days (*p* < 0.05; Fig. 3). Our data also showed that the control group had a significant increase in plasma CORT levels compared to the saline-treated group. Thus, the SPS process was confirmed to cause memory impairment in rats and this procedure was thus used to develop a rat model of PTSD.

Furthermore, the administration of AC (100 mg/kg) attenuated the increase in plasma CORT concentrations in SPS-treated animals (*p* < 0.05). This result indicates that AC reversed the SPS-induced rise in plasma CORT concentrations. The SPS process also induced memory deficits in the rats. Suppression of the increase in plasma CORT concentrations by AC also provides support for the speculation that SPS-caused memory deficits in rats.

### Effects of AC on SPS-induced memory deficits

**Fig. 4:**
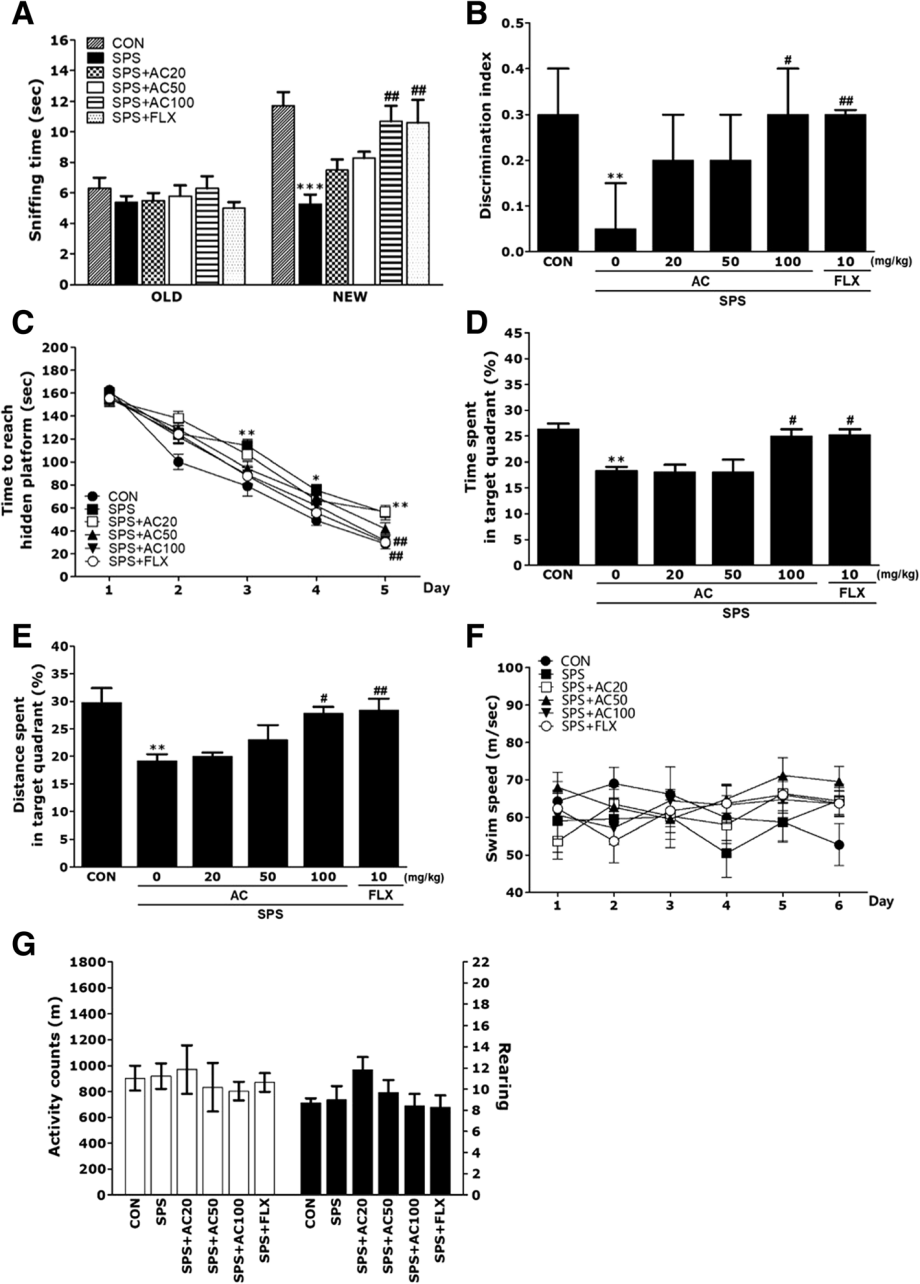
The effects of AC on recognition memory were assessed using a novel ORT. The time spent sniffing familiar and novel objects during a 3-min choice trial (**a**) and the ability to discriminate (**b**) between familiar and novel objects were measured. The MWMW test was used to assess the effect of AC on spatial learning and memory. The time taken to escape (latency) from the water during acquisition trials featuring a submerged platform (**c**), the spent in the target quadrant (**d**), distance travelled in the target quadrant (**e**), and swimming speed (**f**) were the outcome measures. The OFT was to assess the effect of AC on locomotor activity and the total number of rearing behaviors (**g**) (*n* = 6~7/group). *p < 0.05, **p < 0.01, ***p < 0.001 vs. the CON group, #*p* < 0.05, #*p* < 0.05 vs. the SPS group

Object recognition memory, as measured by sniffing behavior directed toward novel and familiar objects, was evaluated in the ORT, and the discrimination index score was estimated (Fig. 4a and b). A significant reduction in the sniffing of novel objects following SPS induction was found compared to the saline-treated group (*p* < 0.001; Fig. 4a). However, in the SPS + AC100 group, the rats showed increased sniffing behavior toward novel objects compared to the SPS group (*p* < 0.01). Discrimination index data also showed significant differences among the groups, with lower discrimination index scores in all SPS, treated groups compared to the controls (*p* < 0.01; Fig. 4b). However, the rats in the SPS + AC100 group showed higher discrimination index scores compared to the SPS group (*p* < 0.05). This also indicated that the recognition memory the SPS + AC100 group was virtually equivalent to that of the SPS + FLX group.

PTSD also affected acquisition trial performance. The SPS group showed significantly longer latencies compared to the saline-treated group throughout the 21 days of testing (Fig. 4c). The SPS + AC100 group showed significantly lower latencies compared to the SPS group (*p* < 0.01 on days 3 and 5, *p* < 0.05 on day 4). Concerning the retention test performed on day 6, the results also indicated that the SPS + AC100 group spent more time in the area around the platform than the SPS group (*p* < 0.05; Fig. 4d and e). Furthermore, the swimming latency of the SPS rats treated with 100 mg/kg AC was comparable to that of rats treated with 10 mg/kg FLX. During training, the average swimming speed of rats was similar among all groups, which suggested that all groups had normal sensory-motor function and survival motivation (Fig. 4f). It also indicated that the escape latency (or swimming latency time) in rats treated with 100 mg/kg of AC was better than that of rats treated with 20 or 50 mg/kg AC, and was virtually equivalent to the rats that received 10 mg/kg of FLX.

We next investigated whether AD could improve cognitive deficits after the SPS procedure. PTSD-related differences in OFT performance were not reflected in locomotor activity (motor function) or the total number of exploration activities (rearing)(Fig. 4g). The SPS procedure did not significantly affect spontaneous locomotion or the total number of rearing behaviors in the OFT. Moreover, AG administration had no effect on spontaneous locomotor activity, irrespective of the dose, or on the total number of rearing behaviors in control rats in the OFT.

### Effects of AC on SPS-induced alterations in the hippocampal CREB and BDNF

**Fig. 5:**
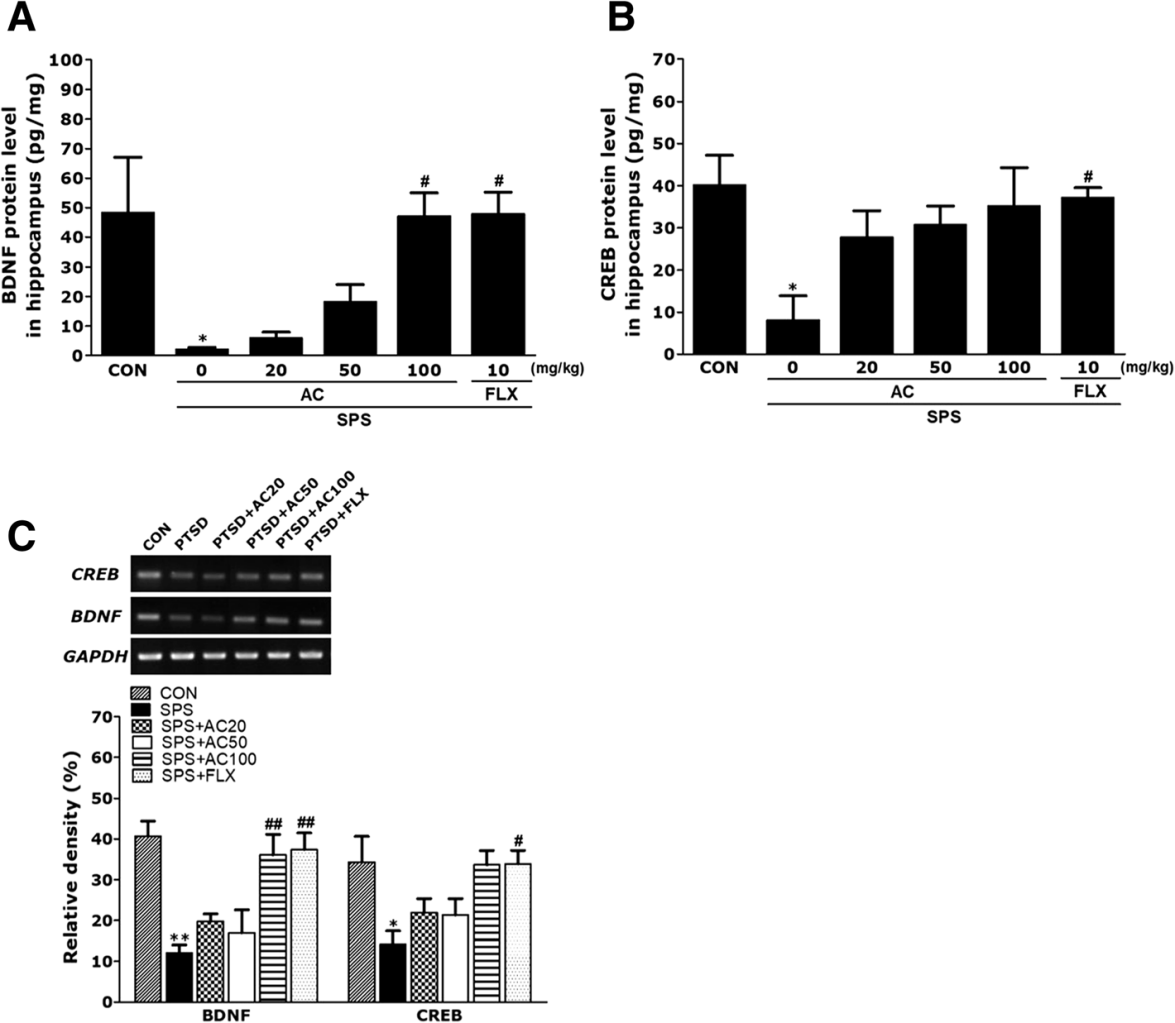
Effects of AC on brain-derived neurotrophic factor (BDNF) and cAMP-response element-binding protein (CREB) protein levels (**a** and **b**) and mRNA expression in the hippocampus of rats with SPS-induced memory impairment. Polymerase chain reaction (PCR) bands on agarose gels and relative intensities are shown in (**c**). The mRNA expression levels of BDNF and CREB were normalized to that of glyceraldehyde 3-phosphate dehydrogenase (GAPDH) mRNA as an internal control (*n* = 4/group). *p < 0.05, **p < 0.01 vs. the CON group, #*p* < 0.05, ##*p* < 0.01 vs. the SPS group

The hippocampal expression levels of BDNF and CREB differed significantly between the groups (Fig. 5). The SPS group showed a significant decrease in BDNF and CREB concentrations compared to the CON group (*p* < 0.05; Fig. 5a and b). Furthermore, treatment with AC significantly increased the magnitude of the decrease in BDNF expression in the hippocampus compared to the untreated SPS group (*p* < 0.05). In addition, the BDNF level in the hippocampus of rats administered 10 mg/kg of FLX was comparable to that of rats treated with 100 mg/kg of AC.

RT-PCR was used to investigate the effect of AC on the expression level of neurotrophic factors in the rat hippocampus that were damaged by PTSD, including the mRNA level of BDNF and CREB proteins. The mRNA levels of BDNF and CREB in the SPS group were significantly lower than those of the CON group (*p* < 0.05 and *p* < 0.01, respectively; Fig. 5c). The decreased expression level of BDNF mRNA in the SPS group was significantly reinstated to levels comparable to those in the CON group after treatment with 100 mg/kg of AC (*p* < 0.05). Our results revealed that BDNF expression in the hippocampus of rats administered 100 mg/kg of AC was comparable to that of rats that received 10 mg/kg of FLX. However, the decrease of BDNF expression in the hippocampus of rats treated with 100 mg/kg of AC was greater than in rats treated with 20 or 50 mg/kg of AC. Investigation of dose-dependent effects of AC showed that 100 mg/kg of AC was the most effective dose for preventing poorer performance in SPS-treated mice on the MWM and ORT tests.

### Effects of AC on hippocampal expression of TNF-α and IL-6 in SPS-treated rats

**Fig. 6:**
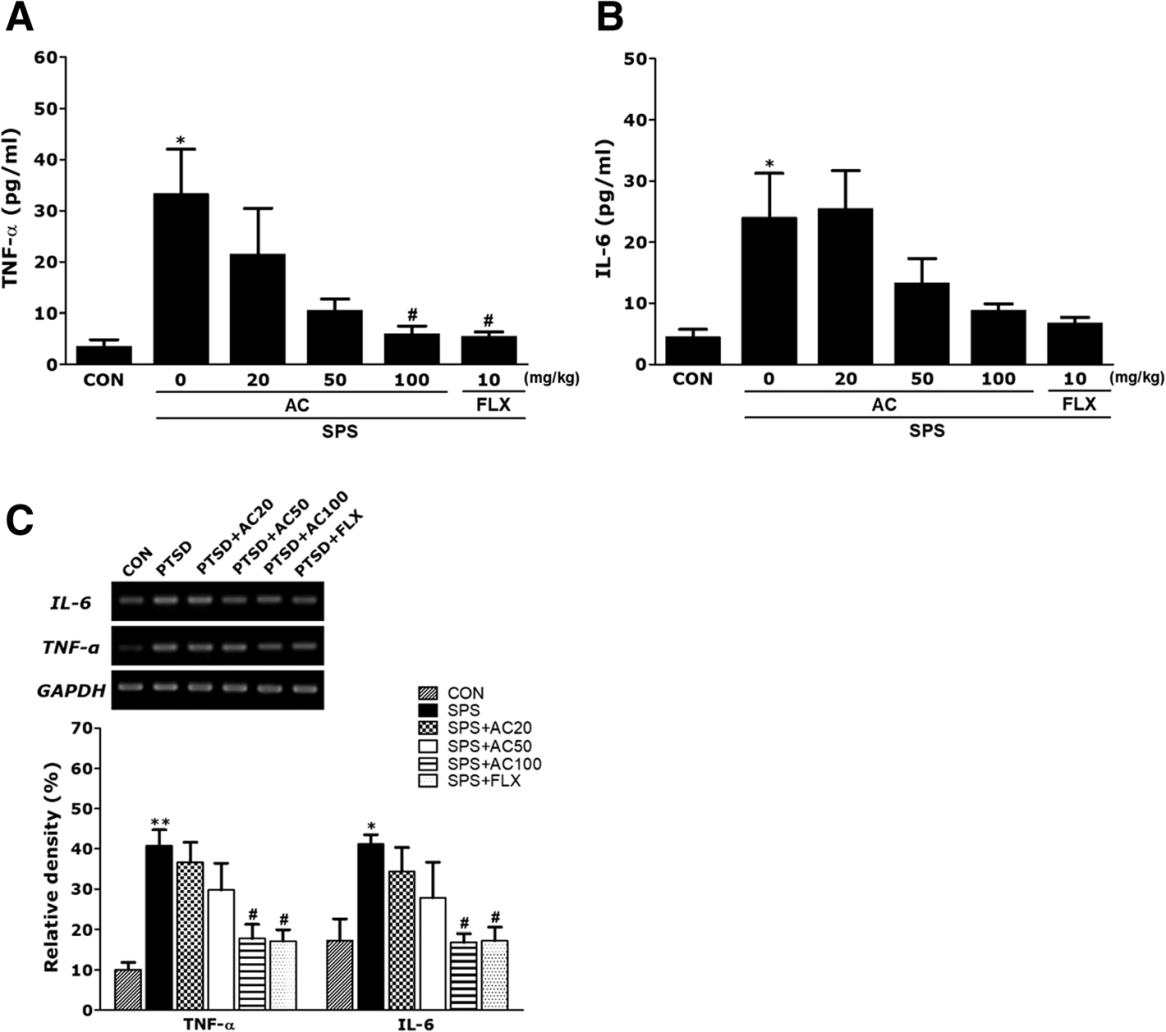
Effects of AC on tumor necrosis factor-α (TNF-α) and interleukin-6 (IL-6) protein levels (**a** and **b**) and mRNA expression levels in the hippocampus of rats with SPS-induced memory impairment. PCR bands on agarose gels and relative intensities are shown in (**c**). TNF-α and IL-6 mRNAs levels were normalized to GAPDH levels as an internal control (*n* = 4/group). *p < 0.05, **p < 0.01 vs. the CON group, #*p* < 0.05 vs. the SPS group

As shown in Fig. 6, SPS produced a significant increase in the levels of TNF-α and IL-6 in rat hippocampus compared to the CON group (*p* < 0.05; Fig. 6a and b). Furthermore, treatment with AC efficiently decreased proinflammatory cytokines to levels comparable to the CON group, in a dose-dependent manner (*p* < 0.05). In addition, the concentration of TNF-α in the hippocampus of rats treated with 10 mg/kg of FLX was comparable to that of rats treated with 100 mg/kg of AC.

The effect of AC treatment on the expression of neurotrophic factors in the hippocampus of rats injured by PTSD was also analyzed, in addition to the TNF-α mRNA and IL-6 mRNA expression levels. The levels of TNF-α mRNA and IL-6 mRNA in the SPS group were significantly higher than in the CON group (*p* < 0.01 and *p* < 0.05, respectively; Fig. 6c). Additionally 100 mg/kg of AC restored TNF-α and IL-6 mRNA in SPS group animals to levels comparable to those of the CON group (*p* < 0.05). The TNF-α and IL-6 mRNA expression levels in the hippocampus of rats treated with 100 mg/kg of AC were comparable to those of rats treated with 10 mg/kg of FLX.

### Effects of AC on treatment on BDNF in the hippocampus of SPS-treated rats

**Fig. 7:**
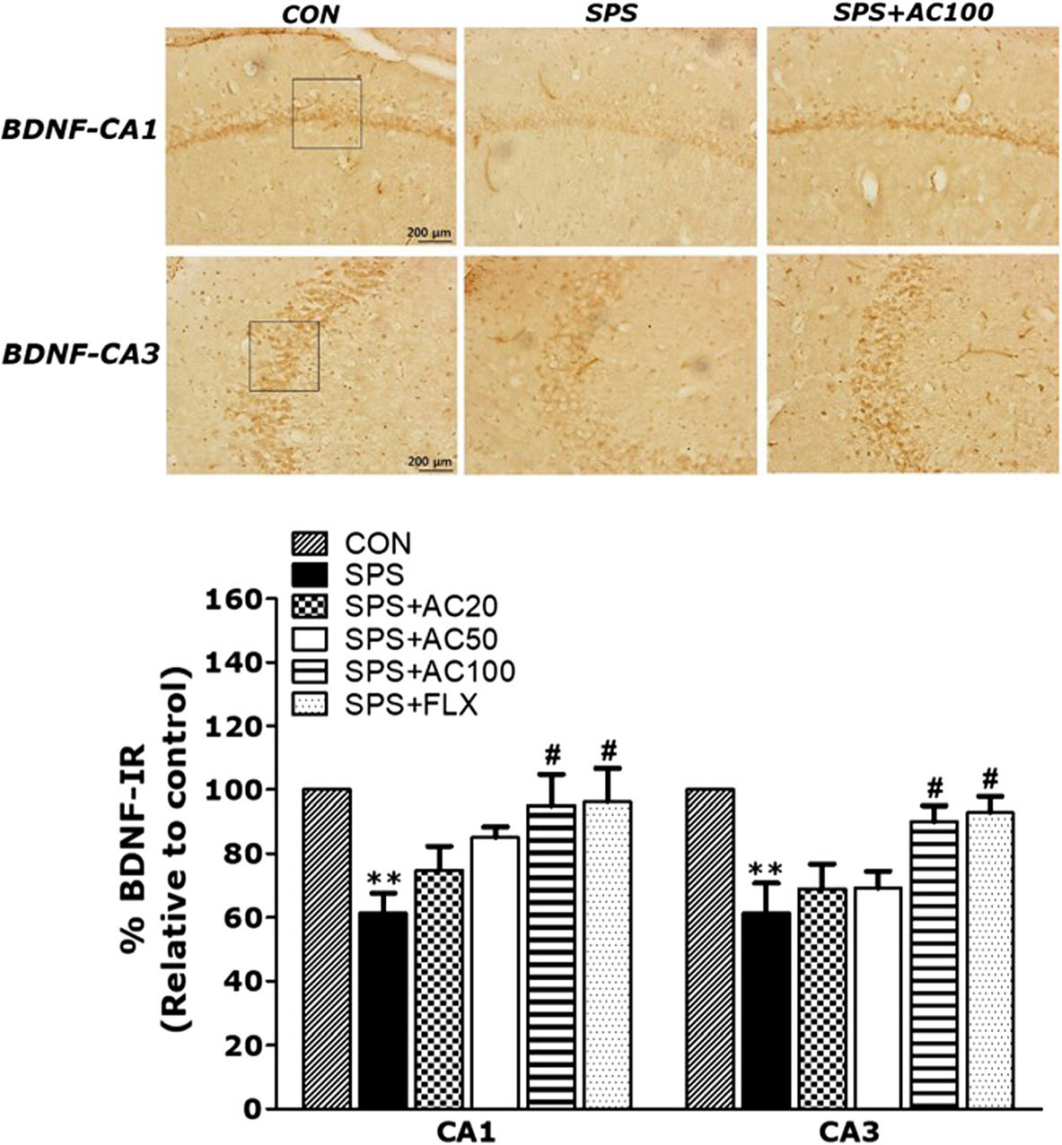
Effects of AC on the mean number of BDNF-stained hippocampal areas after the MWM test. Representative photographs and relative percentages are shown. The scale bar represents 100 μm (*n* = 2~3/group). **p < 0.01 vs. the CON group, #*p* < 0.05 vs. the SPS group

BDNF-like immunoreactivity was primarily observed in the bodies of hippocampal cells (Fig. 7). The density of BDNF immunoreactive fibers in the CA1 and CA3 hippocampal areas of the SPS group decreased by 61.35 and 61.39%, respectively, compared to the CON group. Furthermore, the number of BDNF-like immunoreactive cells was significantly different among all six groups. The SPS-treated rats showed a gradual reduction in BDNF expression in the CA1 and CA3 hippocampal areas compared to the controls (*p* < 0.01) However, AC treatment prior to PTSD increased the number of BDNF-like immunoreactive cells to 83.06 ± 8.55 (95.06 ± 9.79%), compared 60.81 ± 5.22 (61.35 ± 6.23%) in the CA1 hippocampal area of rats with PTSD (*p* < 0.05). AC administration administration prior to PTSD increased the number of BDNF-like immunoreactive cells to 42.63 ± 6.49 (61.39 ± 9.35%), compared 53.00 ± 5.77 (68.66 ± 7.48%) in the CA3 hippocampal area of rats not administered AC (*p* < 0.05).

## Discussion

Our results showed that the memory deficits caused by SPS were associated with severe cognitive impairment, indexed by decreased performance in cognitive function tests. This was reflected in decreased BDNF levels in the hippocampus and an increase in the levels of proinflammatory cytokines. Our results also showed that AC treatment mitigated SPS induced behavioral and biochemical abnormalities while also exerting an anti-inflammatory effect.

In the present study, KA and CA levels in the AC samples were valuable for classifying samples, via HPLC analysis, obtained from various geographical regions in terms of the efficacy of Korean traditional medicine. In a preliminary study, we also determined the optimal ethanol concentration in the solvent. This concentration was used for preparation of AC extract using HPLC analysis, as described previously [29].

SPS induction in our preclinical rat model of PTSD was useful for assessing the core etiological factors of the disorder, including neuroplasticity, and maladaptive cognitive processes, and increased dysregulation in the HPA axis [33]. We subjected rats to SPS to mimic the physiological and mental stress seen in PTSD, and then treated them with AC. We developed the animal model of PTSD and chronic stress based on previous findings that showed that SPS produced memory impairments [34]. In this model, elevated CORT decreased memory ability, which may be related to symptoms that occur following the onset of a traumatic stress disorder [30]. AC restored plasma CORT to normal levels during the 3-week treatment period, suggesting that AC modulated the stress-related dysfunction in the HPA axis, and the behavioral responses, while inhibiting BDNF expression and inflammatory activity. Our results may help to reveal the biochemical mechanisms underlying the effects of AC on the hippocampus, and the behavioral alterations that are caused by low serum levels of CORT.

To examine the effects of AC on recognition and spatial learning, we used ORT and MWM, respectively. We found that SPS caused memory recognition deficits, consistent with our previous study [11]. Furthermore, cognitive impairment was substantially greater after the SPS procedure, as indicated by a significant increase in time spent exploring familiar objects, reduced exploration time for new objects, and a decrease in the discrimination index. These findings showed that the brain is severely affected by PTSD, reflected in decreased recognition and episodic memory [35].

The SPS procedure significantly reduced the time spent sniffing novel objects, and also reduced discrimination index scores, but these deficits were improved by treatment with AC.

As a hippocampus-dependent memory task, the MWM test is commonly used to evaluate cognitive impairment, and to test permanent and reference memory in rodents [34]. In this study, rats subjected to traumatic stress had substantially longer escape latencies (i.e., time taken to reach the platform) than non-stressed rats. Additionally, SPS rats showed impairment in spatial learning in the MWM test. However, AC treatment of SPS rats resulted in more rapid learning of the platform position and shorter escape latencies relative to the untreated rats. Furthermore, AC reversed behavioral abnormalities and reinstated spatial learning and memory in the stressed rats. These results are similar to those seen with prolonged treatment with the antidepressant fluoxetine [33]. Thus, our findings confirm the hypothesis that AC improves the spatial memory impairments caused by traumatic stress.

We applied the OFT to rule out motor deficits, as a potential confounder that could affect task performance. However, significant differences in locomotion and motor activity were not seen among the groups, suggesting that AC treatment did not affect sensorimotor ability. Thus, the improved performance in the MWM test was due to memory ability rather than to differences in activity levels or psychomotor function.

A study by Yang et al., (2015) reported that AC can pass through the blood-brain barrier (BBB) and thus enter the brain [36]. As such, we assume that AC can be widely distributed in rat hippocampus, brainstem, cortex and cerebellum without targeting particular regions in the brain. This is in agreement with results showing that AC has a wide range of pharmacological effects on the central nervous system (CNS). Because it can cross the BBB, AC inhibited the stress-related dysfunction seen in the HPA axis and corrected aberrant behavioral responses.

To identify additional AC-related mechanisms underlying memory improvements, we investigated the effect of AC on BDNF expression in the hippocampus. BDNF is believed to play a critical role in the development of memory. Previous studies have shown that SPS induced memory impairments are associated with substantial reductions in the expression level of BDNF mRNA in the hippocampus, resulting in poor performance on hippocampus dependent tasks [2, 33]. We found that AC treatment significantly reduced the SPS-induced decrease in BDNF mRNA expression, suggesting that the positive effects of AC are mediated by increased BDNF expression and may be associated with an increase in neuronal activity and performance on memory tasks. This suggests that AC normalizes behavioral and neurochemical responses by affecting the secretion of CORT, which in turn implies that AC improves the dysregulation of the HPA axis. The correlation observed between BDNF expression in the brain and improved memory capacity showed that CORT is important in the regulation of cognitive processes associated with BDNF expression, where treatment with AC improved memory. Accordingly, our results suggest that the positive effects of AC may be due to attenuation of dysregulation and restoration of hyperactivity in the HPA axis, via decreasing SPS-induced CORT expression in the CNS. Thus, we have shown that AC treatment inhibited the pathophysiology of PTSD in rats, similar to the antidepressant FLX.

Furthermore, we also found that SPS significantly increased hippocampal expression of TNF-α and IL-6, finally leading to chronic neuroinflammation in the brain. The loss of neurons in the hippocampus generally decreases learning ability and is associated with impaired consolidation of declarative memory in humans and animals [33]. A sustained increase in the expression of proinflammatory cytokines after SPS has been directly linked to psychiatric diseases associated with decreased learning ability and working memory [12, 37].

AC persistently decreases TNF-α and IL-6 mRNA levels, which are increased by SPS; this results in the reversal of chronic inflammation and brain dysfunction [33, 37]. According to the inflammation hypothesis, memory deficits in PTSD are also due to chronic inflammation in the brain [33, 37]. We have shown that the anti-inflammatory actions of AC substantially reverse impaired memory and inhibit increases in the expression levels of proinflammatory cytokines like TNF-α and IL-6. In the present study, AC treatment did not affect IL-6 protein levels, however, memory deficits due to SPS are associated with hippocampus-dependent abilities, as well as with a marked increase in IL-6 mRNA expression levels in the hippocampus [38]. In this study, AC treatment significantly reversed the increase in TNF-α and IL-6 mRNA levels induced by SPS, which is among the beneficial effects of AC. These effects were mediated by decreases in TNF-α and IL-6 mRNA expression and may also be reflected in enhanced neuronal function and better performance in memory tasks. Unfortunately, these findings indicate that there is no correlation between gene and protein function.

## Conclusion

Our findings demonstrate that SPS in a rat model of progressive memory impairment, SPS damaged neuronal function and results in cognitive and memory impairments, as showed by performance on the ORT and MWM tests. Gene and protein expression analyses also confirmed these findings. AC treatment was able to significantly attenuate SPS-induced deficits, as shown by improved cognition, memory and behaviors, increased BDNF levels, and normalization of the HPA axis. Moreover, AC suppressed the increased mRNA expression levels of the proinflammatory cytokines TNF-α and IL-6 in the hippocampus. Therefore, AC may be helpful for reversing the neuronal effects associated with the progressive memory impairments that characterize PTSD.

## Abbreviations

ABC: Avidin-biotin-peroxidase complex
AC: *Aralia continentalis*
ANOVA: Analysis of variance
BDNF: Brain-derived neurotrophic factor
CORT: Corticosterone
CREB: cAMP-response element-binding protein
DAB: 3,3′-diaminobenzidine
ELISA: Enzyme-linked immunoassay
FLX: Fluoxetine
GAPDH: Glyceraldehyde-3-phosphate dehydrogenase
HPA: Hypothalamic-pituitary adrenal
IL-6: Interleukin-6
LPS: Lipopolysaccharide
MWM: Morris water maze
NO: Nitric oxide
OFT: Open field test
ORT: Object recognition task
PBS: Phosphate-buffered saline
PTSD: Post-traumatic stress disorder
RT-PCR: Reverse transcription**-**polymerase chain reaction
SD: Sprague-Dawley
SPS: Single prolonged stress
TNF-α: Tumor necrosis factor-α
